# Supplementary material for: Healthy lifestyle during pregnancy: Uncovering the role of online health information seeking experience
Source: PLoS One. 2022 Aug 1;17(8):e0271989. doi: 10.1371/journal.pone.0271989 (PMC9342740; doi:10.1371/journal.pone.0271989)
Supplement: Attachment — Submitted filename: Response to Reviewers.doc [file pone.0271989.s003.doc]

**Response to reviewers**

**Dear editor:**

Hi and hope to be well; with thanks to the editors and reviewers of our submission, all the reviewers’ comments were carefully revised as followings. If, any more revisions will be needed we will ready to do.

**Reviewer #1:** All comments have been addressed

**Reviewer #2:**

**INTRODUCTION:**

Comment: Line 76-78 should be recanted.

Response: Thanks, it was done.

Comment: The sentence in lines 148-150 does not seem complete.

Response: Thanks, It was done.

Comment: In lines 157 -159, grammatical error should be corrected.

Response: Thanks, it was corrected.

**METHODS:**

Comment: The statement in lines 171-172 is not clear (who had referral; do the authors mean who were referred?). If so, this is at variance with the information in lines 176-177 which states that participants were recruited from 2 heath centres, hence the authors should clarify how and were the participants were recruited.

Response: Thanks, It was done.

Comment: If the sample size of the study was 194 (line 187), why did the authors distribute 210 questionnaires (line 193).

Response: Given that response rate is not always 100% and all the distributed questionnaires are not returned completely, we distributed 210 questionnaires (more than the needed sample size) to achieve the target of 194 completed ones.

Comment: It is not clear why the statement in lie 197 is in the methods section.

Response: Thanks, the sentence was moved to the results section.

Comment: Line 199, what do the authors mean by valid questionnaires are they referring to validated questionnaires?

Response: Yes it is true, the correction was done.

Comment: Lines 201-204 and 219-220 should be referenced.

Response: It was done (the references number 32 and 33)

Comment: Lines 238-239, where is the institution that granted the authors ethical approval for conduct of the study located?

Response: It was added.

**RESULTS:**

Comment: Lines 243-244 is not clear, what's the difference between some University degrees and a bachelor degree?

Response: We used some university degree for Associate degree, it was corrected.

**DISCUSSION:**

Comment: The is no sub-heading titled discussion. It is not clear, whether the information after table 5 is a continuation of the results.

Response: Thanks, it was corrected.

Comment: Lines 386-398- In the whole of this portion, the authors are just citing other studies and not discussing their findings and comparing them to others. This is just lengthening the discussion, hence this portion can be deleted.

Response: It was done.

**With best regards**;

Correspondence
